# Congenital cutaneous aplasia of the limbs: A case report

**DOI:** 10.1016/j.amsu.2022.103496

**Published:** 2022-03-09

**Authors:** Fatima Amaaoune, Radia Chakiri

**Affiliations:** Department of Dermatology, Faculty of Medicine and Pharmacy, Ibn Zohr University, Agadir, Morocco

**Keywords:** Aplasia cutis congenita, Malformation, Skin, Management

## Abstract

**Introduction:**

Congenital skin aplasia, or aplasia cutis congenita (ACC), is a rare congenital anomaly. The incidence is estimated to be between 0.5 and 1 in 10,000 births. We report an observation of ACC of the limbs in a female newborn at D3 of life.

**Case report:**

Female newborn at D3 of life, born at term by vaginal delivery with a birth weight of 2, 900kg. The general examination revealed a hemodynamically and respiratorily stable and apyretic newborn. The dermatological examination noted the presence of translucent exulcerations on both wrists, the inner side of the left knee, and the distal end of the left leg. In view of this clinical aspect, the diagnosis of ACC was evoked. The management was to hospitalize the newborn in a neonatology unit, do a biological check-up with a *trans*-fontanelle ultrasound, echocardiography, and abdominal ultrasound, associated with daily care.

**Discussion:**

Many hypotheses have been put forward to explain the pathophysiological mechanism of CCA, whether isolated or associated with other anomalies. However, at present, the origin of this malformation remains unknown. There is no unambiguous management in the initial phase, as it depends on the type of CCA.

**Conclusion:**

Because of the different possible clinical presentations and existing syndromic associations, it can be thought that it corresponds to a phenotypic expression of various origins, which may be interrelated.

## Highlights

• Congenital skin aplasia, or aplasia cutis congenita (ACC), is a rare congenital abnormality.
• The malformation is characterized by a localized absence of the various components of the skin.
• It can affect the entire skin surface.
• Management of Congenital skin aplasia is controversial, but most cases heal spontaneously within a few weeks with a conservative approach.

## 1 Introduction

Congenital skin aplasia, or aplasia cutis congenita (ACC), is a rare congenital abnormality characterized by a localized absence of the various components of the skin including the epidermis, dermis, and subcutaneous fat [1,2]. The aplasia is located on the vertex in about 86% of cases.

The disorder is most often sporadic but familial cases have been reported [1,2]. Involvement of other parts of the body is possible, especially in association with the vertex. Isolated CCA of the trunk, abdomen, or extremities is very rare. It may also be associated with underlying malformations, such as limb or skull defects, or organ involvement, including the central nervous system, cardiovascular system, and gastrointestinal system [3,4]. Treatment of CCA is controversial, but most cases heal spontaneously within a few weeks with conservative treatment [[3], [4], [5]]. We report here a presentation of CCA with multiple skin lesions on the limbs in a female neonate on the third day of life.

## 2 Case report

We presented a female newborn at D3 of life (Figure), born at term by vaginal delivery with a birth weight of 2, 900kg.

**Figure:**
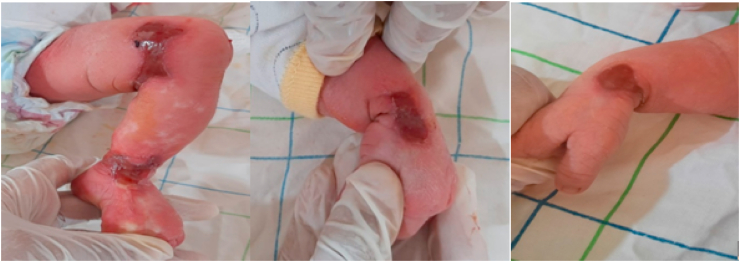
Congenital cutaneous aplasia of the limbs in a female neonate at D3 of life.

The general examination revealed a hemodynamically and respiratory stable and apyretic newborn. The dermatological examination noted the presence of translucent exulcerations, revealing the subcutaneous vessels, well limited, irregular edges, bleeding on contact, fibrinous and crusty in places, located on the two wrists, the inside of the left knee, and on the distal end of the left leg opposite the medial malleolus.

The management involved hospitalizing the newborn in a neonatal unit, carrying out a biological check-up, in particular, a blood count and a CRP, with *trans*-fontanelle ultrasound, echocardiography, and abdominal ultrasound, combined with daily care with 0.9% saline, fatty tulles, and a copper and zinc-based healing cream.

A follow-up of the newborn at the outpatient department was proposed to direct the healing of the lesions. The consent of the patient's parents was obtained for the publication of the case.

This work has been reported in line with the SCARE 2020 criteria [17].

## 3 Discussion

CCA is a rare congenital anomaly occurring in 0.5–1/10,000 live births [3]. Many hypotheses have been put forward to explain the pathophysiological mechanism of CCA, whether isolated or associated with other anomalies. However, at present, the origin of this malformation remains unknown. Due to the large variability in CCA expression and the anomalies that may be associated, it would appear that this phenotype is the result of several different mechanisms, which may be interrelated [6].

The main hypotheses put forward are non-closure of the neural tube, a mechanical or traumatic origin, vascular, genetic, or drug-induced. However, other theories are also found in the literature, including intrauterine infections, smoking and cocaine use during pregnancy, trauma, and adhesion of the amniotic membrane to the fetal skin [6].

The first description was reported by Cordon in 1767 [7] with involvement of the extremities and then by Campbell in 1826 [8] who described a skin aplasia of the scalp. Frieden's classification [9], established in 1986, distinguishes 9 groups of CCA, divided according to the areas affected, the associated anomalies, and the possible mode of genetic transmission. The typical superficial lesion is a translucent membrane through which the underlying structures can be seen. This membrane is crisscrossed by fine vessels and thickens rapidly after birth, especially when fatty dressings are applied.

Thus, through well-directed wound healing, a good quality budding tissue is quickly obtained, which then allows epidermalization, either spontaneously or with the help of skin grafts. Sometimes, this healing phenomenon began in utero and the lesion visible at birth is already a thickened or even epidermised membrane, which has lost its translucent character. In 86% of cases, CCA presents as a single vertex lesion [6], but all parts of the body can be affected, including the trunk and limbs as in our patient with a lower incidence. The aplasia may be localized or extensive. Similarly, the lesion may be superficial or involve underlying tissues such as muscle, galea, periosteum, or bone. For example, it is estimated that in 15–20% of vertex CCA cases there is underlying bone aplasia [3,10], however, there is no correlation between the size of the skin aplasia and the underlying bone aplasia [11].

Multiple skin involvement in CCA is extremely rare; one retrospective study showed only 3 cases (3.3%) of multiple skin lesions (5 or more) out of a total of 90 CCA cases [12], while another study reported no cases of multiple skin lesions out of a total of 22 CCA cases [13]. Furthermore, the majority of CCA cases present as isolated skin lesions, but skull abnormalities are found in about 30% of cases [11,12].

It is important to consider epidermolysis bullosa congenita (EB) as a differential diagnosis. EB is characterized by extremely fragile skin, extensive bullae, and erosions after minimal trauma [5]. EB can be distinguished from our case based on its chronic course with recurrent relapses and the discovery of genetic abnormalities by genetic study.

There is no unambiguous management in the initial phase. The size, location of the lesions, and associated complications must be taken into account to determine the treatment modalities. A review of the literature has shown that small superficial aplasias (less than 2–3 cm in diameter) are generally well cured with conservative care, such as petrolatum, bacitracin, silver sulfadiazine, or occlusive dressings [[3], [4], [5]]. Surgical treatments, such as skin grafts, local flaps, or skin expansions, are considered in cases of more extensive skin damage with underlying bone involvement. In addition, complications that arise, such as infection, hemorrhage, or electrolyte imbalances, may require surgical correction [[3], [4], [5], [14]]. However, even if the lesions are multiple and extensive, local care may result in re-epithelialization if there were no initial CCA-related complications [[4], [15], [16]]. If trisomy 13 is suspected, the diagnosis should be confirmed with a karyotype to orientate management and avoid heavy treatment in these children whose prognosis is very poor. Our patient had multiple skin lesions with no other associated systemic involvement and was treated conservatively with directed wound healing.

## 4 Conclusion

Aplasia cutis congenita is a rare malformation, which can affect the entire skin surface and is most often isolated. However, because of the different possible clinical presentations and existing syndromic associations, it can be thought that it corresponds to a phenotypic expression of various origins, which may be interrelated.

## Provenance and peer review

Not commissioned, externally peer reviewed.

## Ethical approval

Written informed consent was obtained from the patient for publication of this case report and accompanying images. A copy of the written consent is available for review by the Editor-in-Chief of this journal on request.

The consent of the patient's parents was obtained for the publication of the case images. A copy of the written consent is available for review by the Editor-in-Chief of this journal on request.

## Sources of funding

The authors declared that this study has received no financial support.

## Author contribution

Fatima amaaoune: Corresponding author writing the paper.

Radia chakiri: Correction of the paper.

## Registration of research studies

1. Name of the registry:

2. Unique Identifying number or registration ID:

3. Hyperlink to your specific registration (must be publicly accessible and will be checked):

## Guarantor

Fatima amaaoune.

## Consent

Written informed consent was obtained from the patient for publication of this case report and accompanying images. A copy of the written consent is available for review by the Editor-in-Chief of this journal on request.

The consent of the patient's parents was obtained for the publication of the case images. A copy of the written consent is available for review by the Editor-in-Chief of this journal on request.

## Declaration of competing interest

Authors of this article have no conflict or competing interests. All of the authors approved the final version of the manuscript.
